# Allergenicity to worldwide invasive grass *Cortaderia selloana* as environmental risk to public health

**DOI:** 10.1038/s41598-021-03581-5

**Published:** 2021-12-24

**Authors:** Fernando Rodríguez, Manuel Lombardero-Vega, Lucía San Juan, Leticia de las Vecillas, Sofía Alonso, Eva Morchón, Diego Liendo, Marta Uranga, Alberto Gandarillas

**Affiliations:** 1 Allergy Service, Hospital Universitario Marqués de Valdecilla (HUM), Valdecilla 25, 39008 Santander, Spain; 2 CMC R&D Department, ALK-Abelló S.A., Miguel Fleta 19, 28037 Madrid, Spain; 3 Institute for Research Marqués de Valdecilla, Ave Herrera Oria SN, 39011 Santander, Spain; 4 Allergy Service, Sierrallana Hospital, Torrelavega, Spain; 5 Department of Plant Biology and Ecology, University of the Basque Country (UPV/EHU), Bilbao, Spain; 6 Asociación Cantabria Sin Plumeros, Liaño, Spain; 7 INSERM, Occitanie, Montpellier, France

**Keywords:** Biochemistry, Immunology, Ecology, Environmental sciences, Diseases, Risk factors

## Abstract

Allergies to grass pollen affects about 20% of the population worldwide. In the last few decades, the South American grass *Cortaderia selloana* (CS, Pampas grass) has expanded worldwide in a variety of countries including the USA, Australia and Western Europe. In many of these locations, CS has strikingly spread and has now been classified an invasive species. Many pernicious consequences of CS have been reported for local biodiversity, landscape and structures. However, the effect on human health has not been studied. To investigate this issue, we have chosen a European region on the northern cost of Spain where CS spread is overwhelming, Cantabria. We obtained CS pollen extract and analysed the allergenic reaction of 98 patients that were allergic to pollen of local grasses. We determined the skin reaction and the presence of specific IgE antibodies (sIgE) to CS or to a typical autochthonous grass, *Phleum pratense*. We also compared the seasonal symptoms with reported grass pollen counts in the area. The results strongly suggest that CS can cause respiratory allergies at a similar extent to the local grasses. Given that CS pollinises later than the local grasses, this would extend the period of grass allergies in the region for about three months every year, as stated by most of the patients. This is the first study reported on the effects of the striking expansion of CS on human health. Considering the strong impact that respiratory allergies have on the population, our results suggest that CS can currently constitute a relevant environmental health issue.

## Introduction

Grass pollen is one of the main causes of respiratory allergies worldwide and the first cause in North America and Europe, with estimated 20% of the population affected^1^. *Cortaderia selloana* (CS) is a grass of the Poaceae family, of the Danthonioideae subfamily, commonly known as Pampas grass and native to South America. However, in the last few decades CS was introduced in a wide diversity of countries worldwide including the USA, Australia and Western Europe^2^. In these locations, CS has strikingly spread, and it is classified as an invasive species. Within Europe, France, Great Britain, Portugal and Spain are strongly colonised. The United States Department of Agriculture, in a report of 2014, stated: '*Cortaderia selloana* obtained a relatively high impact potential risk score because it impacts natural, anthropogenic, and production systems'^3^. For this reason, it has been forbidden to commercialise, plant or maintain in a variety of countries. One of such countries is Spain, where CS has intensively spread along the northern cost including the regions of Galicia, Asturias, Cantabria and the Basque Country^4,5–7^. First report mentioning *Cortaderia* in Spain are from 1953 in Cantabria^8^.

The allergic incidence of CS is unknown. CS has been referred to as a danger to autochthonous species, strongly affecting biodiversity and landscape. Moreover, it is sporadically mentioned in some venues and discussion groups as a danger to humans, because of material machinery damage and health, such as cuts due to the sharp nature of its leaves, or allergic reactions in contact with the skin^9–11^. However, despite the striking expansion of the grass in regions where it is not autochthonous, there are no studies on the impact on human health so far reported worldwide.

**Figure 1:**
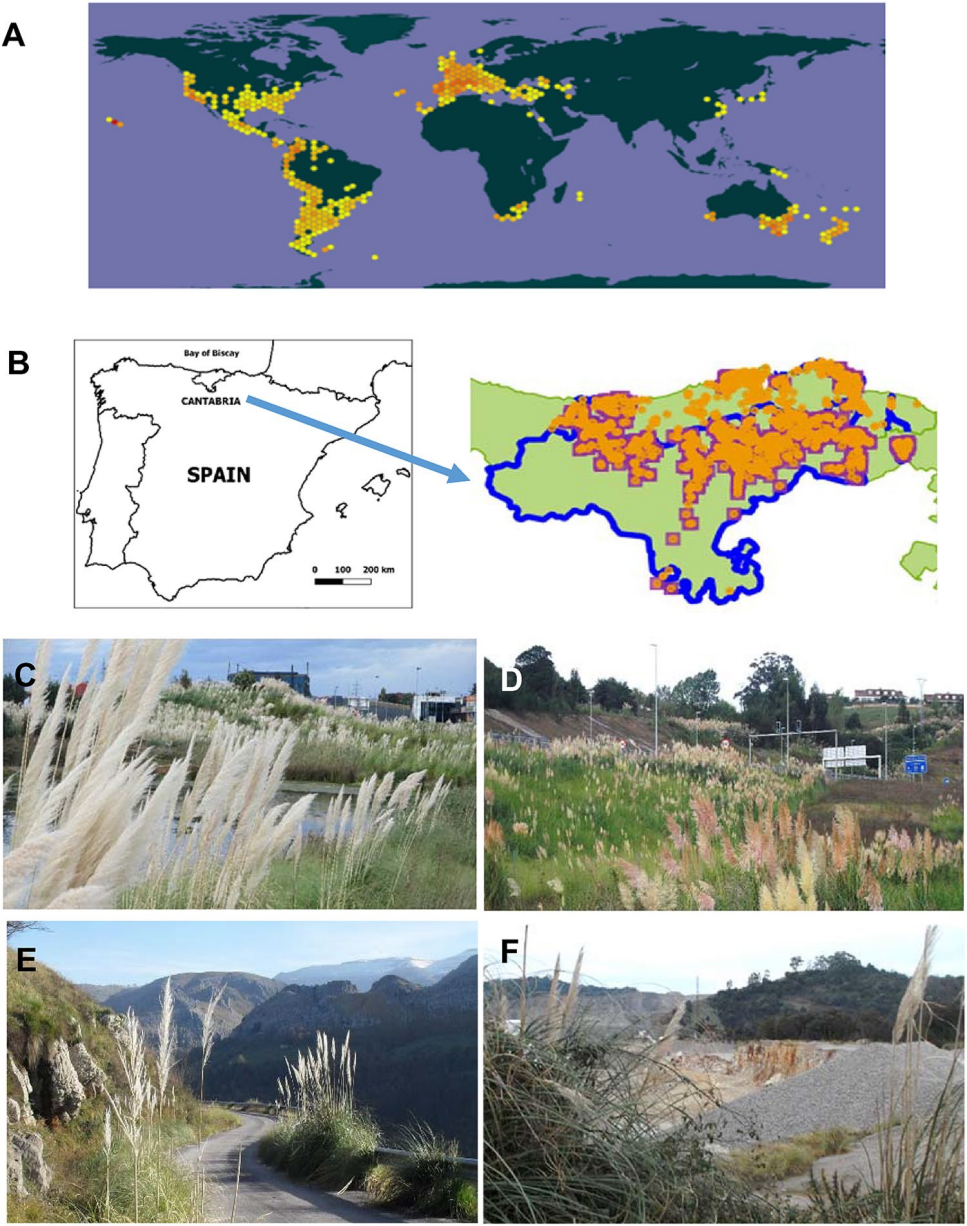
*Cortaderia selloana* (CS) has strongly invaded northern Spain. (**A**) Current spread of CS worldwide (yellow/orange spots). From GBIF.org, *GBIF Home Page*. Available from: https://www.gbif.org/species/2704519. (**B**) Left: location of Cantabria region in Spain (left). Right: current spread of CS in the region is striking, not only on the coast but also inland (blue line). Source: LIFE Stop Cortaderia, http://stopcortaderia.org/language/en/early‐warningnetwork/. (**C**–**F**) Representative photographs of the overwhelming presence of CS in Cantabria region, on the northern coast of Spain. CS has spread near the coast, next to motorways and new house buildings (**C**) but is also notorious inland, next to newly constructed areas (**D**) and even in discrete locations at the mountains, where gravel has been used on small paths (**E**). Stone quarries where the gravel is transported from, are frequently surrounded by CS (**F**).

CS has strongly colonised extensive areas of Cantabria, a typical Northern Spanish region (Fig. 1A,B) of about 500,000 inhabitants, most significantly during 1990–2008, a period of intensive road and house building^6,12^. CS has mainly invaded the coast but it also has reached the inland mountains (Fig. 1B–F). The plant has spread by human activities. It is used in motorways to retain the road slope soil and as a natural barrier^13,14^ and it is transported with construction aggregates and gravel from stone quarries. Therefore, it is consistently found next to roads, new buildings or small paths covered with gravel and is abundant around stone quarries (Fig. 1C–F). Plans for limiting and eradicating the growth of this invasive plant have been debated in the local parliament due to pressure of ecologist organisations although only limited programmes were implemented. Currently, the European Union is funding a regional network for fighting the inland expansion of the grass and diffusing the rapidly increasing problem among the European society (3.5 million euros for 2018–2022^15^). However, the presence of CS on the northern cost of Spain is still overwhelming.

Cantabria is a good paradigmatic territory to investigate the allergenic effects of CS on the human population. In Cantabria, autochthonous grass pollens peak from April to July^1,16,17^, when they cause a concomitant peak of hay fever. It is estimated that the percent of the population suffering from grass-associated hay fever in Cantabria is about 19% of patients diagnosed of rhinoconjunctivitis and 14% of asthmatic patients^18^. In contrast to the autochthonous grasses, CS in the North of Spain flourishes from mid August to October ^5^. Grass pollens of the Pooideae subfamily, the main grasses found in temperate climates of the North Hemisphere, contain proteins with similarities in their antigens^19,20^. We questioned whether patients allergic to the autochthonous grass pollen (*Phleum pratense*, Phl, as representative species) might also be allergic to CS pollen. Allergenic molecules of groups 1 and 5 (Phl p1 and Phl p5) are main antigens inducing allergies due to their high capacity to bind to immunoglobulin IgE of the human immune system. To investigate this issue, we analysed the skin reaction to Phl and CS extracts of 98 patients of Cantabria that were allergic to local grass pollen. In addition, we determined the presence of specific IgE antibodies (sIgE) to Phl and CS pollen extracts and to the single allergens Phl p 1, Phl p 5, Phl p 7 and Phl p 12 in blood serum. We also compared the seasonal symptoms with reported grass pollen counts. The results very strongly suggest that CS is a significant cause of respiratory allergies, at a similar extent as the local grass. This might thus extend the period of respiratory allergies in the region for more than three months every year. This is the first study reported on the effects of the striking expansion of CS on human health and it has implications in all the regions of the world where CS has become a widespread invasive grass. Considering the implications that respiratory allergies have on health, not only by the direct effects but also by allowing opportunist infections, our results suggest that CS can constitute a significant public health issue. This risk must be added to the ecological impact, in order to encourage efforts for eradicating CS from invaded, non-autochthonous regions.

## Materials and methods

### Setting

This study was conducted in Cantabria, a region of the North coast of Spain.

### Design and patients

A cross-sectional study with prospective data collection was performed at the Allergy Services of the Marqués de Valdecilla University Hospital in Santander and the Sierrallana Hospital in Torrelavega (Cantabria, Spain).

98 patients diagnosed of rhinoconjunctivitis, asthma or both, caused by sensitization to grass pollen, were included in a sequential way from October 2015 to March 2016.

Written informed consent was obtained from all patients before entering the study. The study met the principles of the 1975 Helsinki declaration and was reviewed and approved by the local Research Committee of Cantabria (CEIC reference number 2015.207).

A serum sample was obtained from each patient and stored at – 20 °C until used.

### Pollen extract preparation

All methods were performed in accordance with the relevant guidelines and regulations.

*Cortaderia selloana* (CS) pollen was obtained commercially (Iber-Polen, Jaén, Spain) and then extracted at a 1:10 (w/v) ratio in PBS pH 6.5 with magnetic stirring for 90 min. at 5 °C. The soluble fraction was separated by centrifugation. After dialysis against PBS, the extract was filtered through 0, 22 µm filters. Protein content was determined by Bradford method (BioRad, Hercules, CA, USA). Two different batches were obtained (07 and 09) with consistent results.

Part of the extract was adjusted to 0.25 mg protein/ml and formulated in PBS with 50% glycerol, phenol 0.51% (SPT buffer). The remaining extract was stored in aliquots at − 20 °C.

*Phleum* *pratense* (Phl) pollen extract was made as described for CS. The origin of the pollen in this case was ALK Source Materials, Post Falls, Idaho, USA.

The protein profiles of the CS or the Phl extracts were determined by polyacrylamide electrophoresis in the presence of sodium dodecyl sulphate (SDS-PAGE) under reducing conditions (Invitrogen-Novex tricine gels 10–20% acrylamide, Fisher Scientific, SL, Madrid Spain).

### Skin prick test

Patients were skin prick tested (SPT) with a commercial extract (ALK-Abelló, S.A. Madrid, Spain) of Phl and the CS extract. Histamine dihydrochloride solution (10 mg/ml) and SPT buffer were used as positive and negative control (no reaction), respectively.

The SPT wheal areas were measured by planimetry. A cut-off area of 7 mm^2^ (about 3 mm average diameter) or higher was considered a positive test result (histamine).

The CS extract was tested in 10 control subjects, that were not sensitised to grass pollen, with negative result (no reaction).

### IgE assays

Serum samples were tested for IgE antibodies against *Phleum pratense* (Phl) pollen extract and the allergens Phl p 1, Phl p 5, Phl p 7 (polcalcin) and Phl p 12 (profilin) (ImmunoCap FEIA, Thermo Fisher Scientific, Barcelona, Spain).

In addition, specific IgE against Phl and CS pollen extracts was determined by RAST (Radio Allergo Sorbent Test). Paper discs were activated with CNBr and sensitised with the pollen extracts as described by Ceska et al.^21^*.* Phl and CS discs were incubated overnight with 50 µL of the patient’s serum and after washing (0.1% Tween-20 in PBS), with approximately 100,000 cpm of the iodine 125–labeled anti-IgE mAb HE-2 for 3 h as described^22^. Finally, the discs were washed, and their radioactivity was determined in a gamma counter. sIgE values in kilounits per litre were determined by interpolating in a standard curve built up with *Lolium perenne*—sensitised discs and 4 dilutions of a serum pool from patients with grass allergy, which was previously calibrated in arbitrary kU/l.

A cut-off value of 0.35 kU/l was considered positive for both ImmunoCap and RAST. There was a very significant correlation between the sIgE against Phl determined by both methods (r Spearman = 0.8874, p < 0.0001).

### RAST inhibition assay

Paper discs were sensitised as above in the IgE assays section and then incubated with 50 µL of a serum pool from all patients combined. 50 µL of (inhibitory) CS extract solution (in serial dilutions) were added onto the paper discs and incubated overnight at room temperature. All other incubations were performed as indicated above in the IgE assays section. The % of inhibition was determined for each extract dilution by radioactive counts (cpm) and calculated by means of the following equation:

$${1}00 \, \times \, \left( {{1 }{-} \, \left[ {\left( {{\text{cpmx }}{-}{\text{ cpm1}}00\% } \right) \, / \, \left( {{\text{cpm}}0\% \, - {\text{ cpm1}}00\% } \right)} \right]} \right)$$

Cpmx corresponds to the mean radioactivity of the discs incubated with inhibitor at a given X dilution. cpm100% corresponds to the blank control samples of the assay (no serum pool added). cpm0% corresponds to the signal obtained with no inhibitor extract added.

## Results

To investigate whether patients allergic to the local pollen react to CS pollen, we chose a cohort of 98 patients from Cantabria. Table 1 shows the demographic and clinical characteristics of the patients. All of them were diagnosed with rhinitis during the spring season and grass pollen sensitisation. In addition to nasal symptoms, 98% had also associated conjunctivitis, 31.6% suffered from asthma and 8.2% from urticaria. Only 12.2% had food allergies and 2 out of 98 drug allergies. 53.06% of the patients underwent grass pollen immunotherapy. 76.5% of the patients referred living in areas with high presence of CS. 78.6% of patients presented a worsening of their pollen allergic symptoms from August to November (“delayed reactivation”). In addition, 56.12% of the cohort were polysensitised including other pollens such as *Plantago spp.* (18/98), trees (9/98), *Parietaria* spp. (6/98), animal dander (11/98) or house dust mites (38/98).

**Table 1:** Demographic and clinical data and SPT results.

Patient	Age (years)	Sex^a^	Years living in Cantabria	Exposure^b^	Clinical symptoms^c^	Months with symptoms	Other sensitisations^d^	Cutaneous reaction^e^	
								*C. selloana*	*P. pratense*
E0416001	50	M	10	(*)	RC	May–Oct	HDM/plantago	**47**	59
E0416002	38	F	38	(*)	RCA	Mar–Oct	–	**40**	24
E0416003	23	M	23	(–)	RCAU	Mar–Sep	HDM/dog	**30**	21
E0416004	44		10	(*)	RCA	Apr–Sep	HDM	**47**	45
E0416005	46	M	46	(*)	RC	Mar–Oct	HDM/platanus	**10**	22
E0416006	49	M	20	(*)	RC	Jun–Oct	–	**67**	34
E0416007	41	F	41	(–)	RCA	Mar–Aug	Dog	**32**	113
E0416008	27	F	27	(*)	RC	May–Aug	HDM	**31**	65
E0416009	27	F	27	(*)	RC	Mar–Oct	–	**49**	76
E0416010	55	M	55	(*)	RC	Apr/Oct	–	**30**	54
E0416011	50	F	20	(*)	RC	Jul–Oct	–	**35**	38
E0416012	36	M	36	(*)	RC	May–Oct	–	**16**	33
E0416013	20	M	20	(*)	RC	Apr–Aug	HDM	**39**	94
E0416014	42	M	37	(*)	RC	Mar–Sep	–	**19**	92
E0416015	39	M	12	(*)	RCA	May–Oct	HDM	**18**	25
E0416016	45	M	45	(*)	RC	May–Sep	–	**21**	20
E0416017	52	M	31	(*)	RCA	Mar–Jul	–	**63**	92
E0416018	45	M	40	(*)	RC	May–Sep	–	**42**	48
E0416019	34	M	34	(*)	RC	May–Oct	–	**67**	78
E0416020	30	M	25	(*)	RC	Feb–Nov	Cat	**29**	48
E0416021	44	M	21	(*)	RCA	May–Set	–	**111**	162
E0416022	38	M	38	(*)	RC	Apr–Aug	HDM	**7**	37
E0416023	43	M	7	(–)	RC	Feb–Nov	Plantago	**14**	23
E0416024	50	M	50	(*)	RC	May–Set	–	*6(N)*	39
E0416025	33	F	33	(*)	RC	Apr–Oct	–	**49**	28
E0416026	29	F	29	(*)	RC	Apr–Set	–	**37**	77
E0416027	48	M	48	(*)	RC	May–Oct	HDM	**68**	35
E0416028	41	M	4	(*)	RC	Apr–Jun	–	**86**	48
E0416029	42	M	14	(*)	RCU	Apr–Sep	HDM	**21**	48
E0416030	29	M	29	(*)	RC	May–Sep	HDM	**48**	50
E0416031	42	M	42	(*)	RCA	Mar–Aug	HDM	**36**	48
E0416032	48	M	15	(–)	RC	Mar–Aug	0	**49**	69
E0416033	25	M	25	(–)	RC	May–Sep	HDM	**34**	169
E0416034	39	F	15	(*)	RC	Apr–Jul	–	*6(N)*	83
E0416035	53	F	17	(*)	RC	Apr–Nov	–	**27**	38
E0416036	48	M	6	(*)	RC	Apr–Oct	HDM	**61**	33
E0416037	63	F	63	(*)	RC	Apr–Jul	–	**27**	33
E0416038	58	M	58	(*)	RC	May–Aug	–	**23**	22
E0416039	39	M	39	(*)	RC	May–Aug	HDM	**50**	114
E0416040	40	F	40	(*)	RC	May–Oct	HDM	**18**	37
E0416041	31	F	31	(*)	RC	May–Aug	–	*1(N)*	26
E0416042	29	F	29	(*)	RCAU	May–Jul	–	**13**	31
E0416043	32	F	18	(*)	RC	Jul–Sep	HDM	**24**	171
E0416044	42	F	8	(*)	RCA	Apr–Aug	Parietaria	**17**	22
E0416045	42	M	2	(*)	RC	Jul–Sep	–	*1(N)*	37
E0416046	22	F	22	(*)	RCA	Mar–Aug	HDM/parietaria	**79**	64
E0416047	34	M	34	(*)	RCA	May–Oct	HDM/cat	*1(N)*	18
E0416048	39	M	39	(*)	RCA	May–Jul	–	**41**	42
E0416049	41	M	15	(*)	RC	Apr–Oct	HDM	**57**	44
E0416050	28	F	27	(*)	RC	Apr–Nov	HDM	**15**	23
E0416051	30	M	30	(*)	RC	Mar–May	HDM/plantago/cupresaceous/parietaria	**32**	128
E0416052	63	M	63	(*)	RC	May–Oct	HDM	**40**	37
E0416053	22	M	20	(*)	RC	Apr–Aug	Dog	**21**	75
E0416054	32	F	32	(*)	RC	May–Oct	HDM	**22**	67
E0416055	41	F	7	(*)	RCA	May–Sep	HDM/parietaria/plantago	**15**	41
E0416056	44	F	44	(*)	RC	Apr–Nov	HDM/horse/dog/cat	**34**	79
E0416057	23	F	23	(–)	RCA	May–Sep	HDM	**13**	49
E0416058	41	F	41	(*)	RC	Apr–Sep	–	**38**	36
E0416059	31	F	31	(*)	RCAU	Feb–Nov	Cat/dog/plantago/HDM	**8**	15
E0416060	41	M	36	(*)	RC	May–Sep	–	**37**	45
E0416061	29	F	29	(*)	RC	Apr–Sep	–	**29**	68
E0416062	44	M	43	(–)	RCA	May–Jul	Cat	**5**	35
E0416063	50	F	50	(–)	RC	Jun–Nov	HDM	**11**	21
E0416064	26	F	1.5	(*)	RC	Mar–Oct	–	**17**	46
E0416065	69	M	69	(–)	RCA	May–Nov	–	*5(N)*	66
E0416066	39	F	31	(*)	RCA	Apr–Aug	–	**27**	47
E0416067	40	M	40	(*)	RCA	May–Nov	Plantago	**10**	14
E0416068	26	F	26	(*)	RC	May–Sep	Plantago	**34**	39
E0416069	67	M	67	(–)	RC	May–Sep	–	**12**	42
E0416070	70	F	70	(*)	RC	May–Nov	HDM	**48**	46
E0416071	32	F	32	(*)	RC	Apr–Oct	HDM	**20**	20
E0416072	30	F	30	(*)	RC	Apr–Jul		**130**	34
E0416073	18	F	18	(–)	R	May–Jun	HDM/plantago	**13**	26
E0416074	50	M	24	(*)	RC	May–Oct	–	**54**	53
E0416075	35	M	35	(–)	RC	Apr–Aug	–	**75**	77
E0416076	23	M	23	(*)	RCA	Apr–Aug	HDM/plantago	**71**	96
E0416077	38	F	38	(*)	RCA	May–Oct	HDM	**38**	22
E0416078	34	F	34	(–)	RC	Apr–Oct	Parietaria	**37**	57
E0416079	23	M	23	(*)	RC	Apr–Oct	HDM	*1(N)*	24
E0416080	36	M	36	(*)	RCA	Apr–Sep	–	**37**	36
E0416081	32	F	32	(*)	RCA	Apr–Jul	HDM/parietaria	**28**	78
E0416082	36	F	9	(–)	RC	May–Jun	–	**18**	28
E0416083	31	M	31	(–)	RC	Apr–Jul	–	**3**	29
E0416084	23	M	23	(*)	RCA	May–Jul	–	**27**	56
E0416085	39	F	39	(*)	RC	May–Aug	HDM	**67**	55
E0416086	29	F	10	(–)	RCAU	May–Sep	Platanus/cupresaceous/plantago	**11**	39
E0416087	18	F	12	(*)	RCA	Apr–Sep	Plantago	**16**	96
E0416088	46	M	15	(–)	RCA	Mar–Sep	HDM/cat/dog/horse/platanus/cupresaceous/plantago	**32**	32
E0416089	30	F	30	(–)	RCAU	Mar–Jul	Platanus/plantago	**33**	138
E0416090	23	M	23	(–)	RCU	Mar–Oct	–	**18**	52
E0416091	39	F	39	(*)	RCA	Mar–Nov	HDM/plantago	**17**	80
E0416092	20	M	20	(*)	RCU	Apr–Jul	–	**107**	65
E0416093	63	F	63	(–)	RC	Apr–Aug	–	**20**	23
E0416094	23	F	23	(–)	RC	Mar–Sep	Plantago/platanus	**5**	29
E0416095	44	F	26	(–)	RC	Apr–Jul	Platanus/plantago/cupresaceous	**34**	39
E0416096	43	F	43	(–)	R	May–Jun	–	*1(N)*	89
E0416097	37	F	37	(*)	RC	May–Oct	–	**80**	27
E0416098	30	M	30	(*)	RCA	Mar–Jul	Dog/cupresaceous/plantago	**20**	56

*SPT* Skin prick test.

^a^*F* female, *M* male.

^b^Exposure (*) means that the patient lives in an area in which *C.* *selloana* plants have been identified.

^c^*A* athma, *C* conjunctivitis, *R* rhinitis, *U* urticaria.

^d^*HDM* House Dust Mites.

^e^Wheal area (mm^2^). Negative reaction to *C. selloana* is highlighted in italic numbers (N).

Numbers in bold indicate positive reaction (>6).

**Figure 2:**
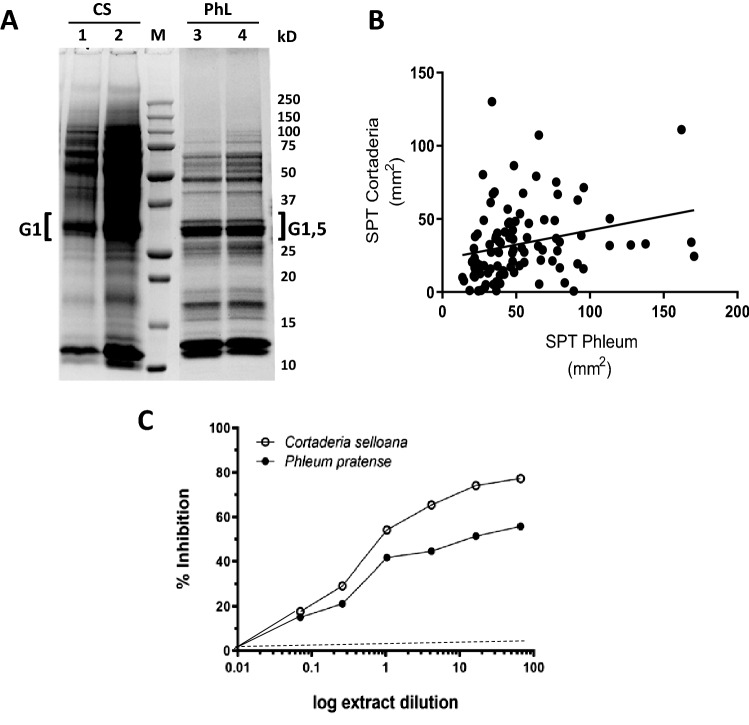
*Cortaderia selloana (CS) pollen* shares antigens and inmmunogenicity with authoctonous grass *Phleum pratense* (Phl). (**A**) SDS‐PAGE profile of CS (lanes 1, 2) or Phl pollen extract (lanes 3,4) extract. Lane 1 and 2 corresponds to 20 µl and 40 µl of CS pollen extract, respectively (see also Supplementary Fig. 3) representative of two independent batches. Lane 3 and 4 correspond to 20 µl from two different batches of Phl pollen extract. M: the molecular weight markers. Brackets indicate the position of the allergenic groups (G) according to the documented apparent molecular weights. (**B**) Correlation between SPT result for PhL and CS Pearson r: 0.2558; R^2^: 0.06543; p value : 0.0110 (two‐tailed). (**C**) IgE Cross‐reactivity of CS and Phl pollen antigens as measured by radioallergosorbent **(**RAST) inhibition assays. Note that if there were no cross‐reaction the Phl plot should be flat to cero (broken line).

CS pollen extract is not commercially available to run skin prick tests or sIgE determination. Therefore, we isolated and prepared a CS pollen extract by a standard extraction protocol used for pollens (see Materials and Methods). The yield protein/pollen was about 50 mg/g, a typical concentration obtained for other grass pollens (our own unpublished data). Grass-specific ELISA assays showed that the CS extract did not contain group 5 antigen, as expected for a non-Pooideae subfamily grass (< 0.3 µg group 5/mL^23^). The profile of the protein extract by SDS-PAGE shows a group of 25–37 kD bands with the mobility of the grass group 1 allergens and it might correspond to the homologous CS group 1 (arrow, Fig. 2A;^19,24^).

Isolated CS pollen extract was used on cutaneous tests on the patient cohort, in parallel with Phl pollen extract, as a representative of the local autochthonous grass pollens. All 98 patients gave a positive response by skin prick test to Phl pollen extract and 89% of the patients were also positive to CS pollen extract (Table 1). Moreover, there was a significant correlation between the area of the papule to Phl and to CS (rPearson = 0.2558, p = 0.01; Fig. 2B). As a control, 10 patients negative for skin reaction to Phl were found also negative for CS extract. These results show a strong coincidence in the cutaneous reaction to CS and to the local grass. To further study the interspecies cross reaction of the patient sera, we run by RAST (radio allergo sorbent test) inhibition assays. As shown in Fig. 2C, Phl extract significantly competed with CS extract to bind the serum sIgE from the patients.

Supplementary Table I displays the results of sIgE masurement. We determined sIgE to Phl and to the allergens Phl p 1, Phl p 5, Phl p 7 and Phl p 12 by ImmunoCap (Thermo Fisher) and to CS by RAST. All patients had serum sIgE to Phl by both ImmunoCap and RAST, in agreement with the skin prick test results. We determined the correlation between both techniques in detecting the sIgE for Ph. The relation was rSpearman = 0.8874, p < 0.0001. Values obtained by RAST were below those obtained by ImmunoCAP (factor = 0.36) and the linear range for RAST (0.17–27) was shorter than for ImmunoCAP (0.35–100). Nevertheless, the correlation between both techniques was good, indicating that the sIgE data obtained by ImmunoCap can be compared with the sIgE data obtained by RAST (Supplementary Fig. 1). All patients but seven contained sIgE specific to CS extract. Interestingly, within the seven patients with a negative sIgE test to CS, 5 displayed a negative skin response to CS and the other 2 displayed a weal smaller than 14 mm^2^. Therefore, there was a strong correlation between the skin response and the sIgE to CS in serum (Fisher's exact test, p < 0.0001; Supplementary Table II).

**Figure 3:**
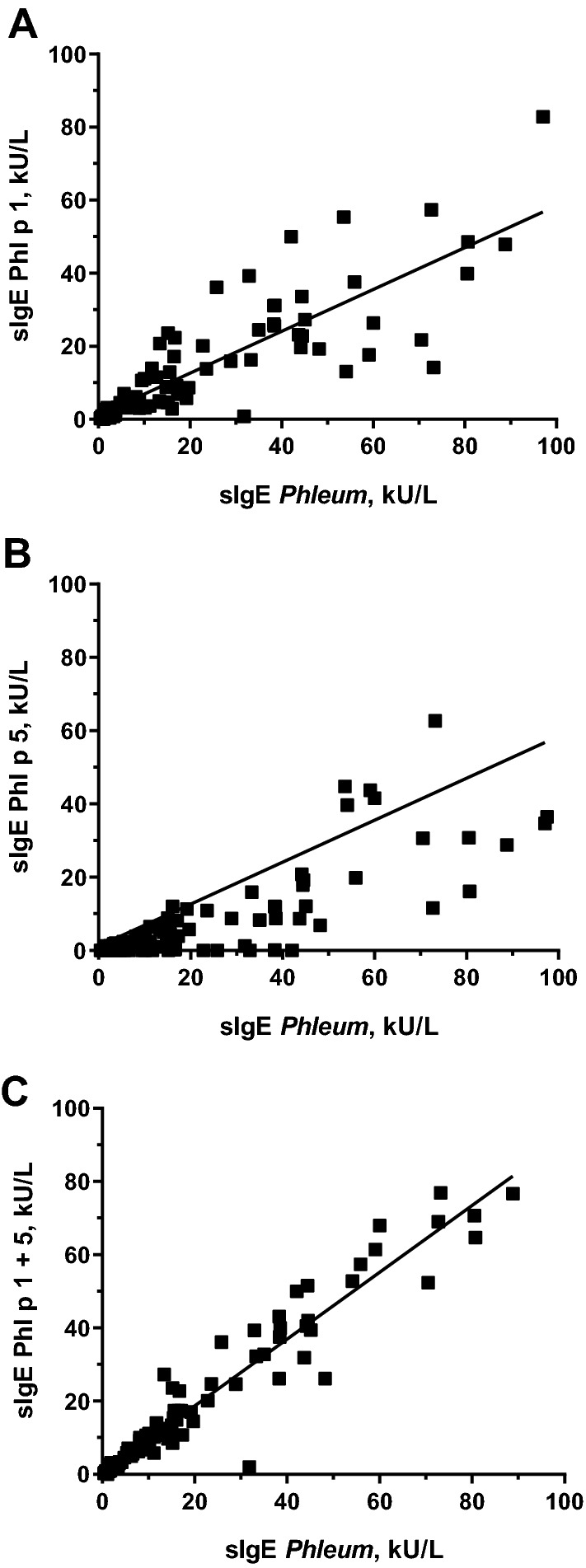
Linear regression of sIgE to *Phleum pratense* whole extract (ImmunoCap) versus sIgE to Phl p 1 (**A**), sIgE to Phl p 5 (**B**) and sIgE to Phl p 1 + Phl p 5 (**C**).

We measured the presence of sIgE to the individual allergens Phl p 1, Phl p 5, Phl p 7 and Phl p 12 in the sera from the patients (Supplementary Table I). For the pan-allergens Phl p 7 (polcalcin) and Phl p 12 (profilin), only 27 patients (27.5%) had sIgE to any of them. Consequently, the patient sensitisation to these allergens cannot explain the high cross-sensitisation to Phl and CS in this group of patients. The prevalence of sIgE to Phl p 1 was very high (98%) and only two patients (# 45 and 83) were negative for IgE to Phl p 1. Consistently, these patients also displayed a negative skin response to CS extract. The prevalence of Phl p 5 was lower but still important (72%). Twenty-seven patients of the cohort displayed no IgE to Phl p 5 in serum. However, of these, only five patients were negative for skin response to the CS extract. There was a significant linear regression between the sIgE to the whole Phl extract and the sIgE to Phl p 1 (Fig. 3A) or Phl p 5 (Fig. 3B). From the slope of the regression line, we can conclude that every allergen accounts for about 50% of the total IgE response to the whole extract, being the IgE-response to Phl p 1 slightly higher. The reaction to Phl p 1 plus Phl p 5 is similar to the reaction to whole Phl extract (Fig. 3C), strongly suggesting that groups 1 and 5 are the main allergens of Phl and they account for most of the IgE to the whole Phl extract. There is a significant correlation between the sIgE to CS extract and the sIgE to Phl whole extract, to Phl p1 or to Phl p5 (Table 2). The correlation is stronger for the whole extract or for Phl p 1 (r = 0.75) than for Phl p 5 (r = 0.55). These results suggest that the common reaction observed in the patients to pollen extract of Phl and CS might reside in the antigenic Group 1 that is ubiquitous in all grasses^25^.

**Table 2:** Correlation between sIgE to *C. selloana* and to Phl p 1 sIgE, to Phl p 5 sIgE and to *P. pratense* sIgE.

	sIgE CS (Ku/L) vs. sIgE Phl p 1 (kU/L)	sIgE CS (Ku/L) vs. sIgE Phl p 5 (kU/L)	sIgE CS (Ku/L) vs. sIgE Phl (kU/L) ImmunoCap
r Spearman	0.755	0.552	0.7476
95% confidence interval	0.6513 to 0.8311	0.3920 to 0.6795	0.6400 to 0.8264
P (two‐tailed)	< 0.0001	< 0.0001	< 0.0001
Significant? (alpha = 0.05)	Yes	Yes	Yes
Number of XY pairs	98	98	96

*Phl p:* *Phleum* antigen group.

We analysed the measured grass pollen concentration along the year in the region. The regional agency Health Department of the nearby Basque Country detected a spring main peak of grass pollen around May and a second, August-to-October peak, in the air of Bilbao, a city 50 km off Cantabria with a similar climate and density of CS. It is interesting that most patients (78.6%) in the study mentioned a second allergic reaction around September–October (Table 1, Supplementary Fig. 2). This indicates a timely correlation between grass pollen and the referred allergic symptoms by the patients.

## Discussion

We could not find in the literature any report on the impact of CS on human health. This is somehow surprising and highlights the need of studies on the issue, considering the widespread presence of this invasive plant worldwide^1–5^. Concerns about the consequences of CS expansion are evident among professionals regarding the impact of CS in ecology, industry or health^9–12^. Our study addresses for the first time the potential allergenic effects of CS pollen. Given the wide impact of grass allergy in the population, this constitutes a public health issue.

We here present several lines of evidence strongly suggesting that patients allergic to pollen of northern Spanish autochthonous grasses, such as Phl, are also allergic to pollen of CS: (i) 89% of the patients allergic to Phl were sensitised to CS, as evident both by skin reaction and by sIgE in serum; (ii) the timely coincidence along the year of allergy symptoms reported by patients, grass pollen counts and flourishing of CS; (iii) the presence in CS of a protein band with a mobility compatible with grass allergenic group 1 and the strong prevalence of this group in the sIgE to Phl.

The high cross-sensitisation to Phl and CS pollen in this cohort of patients is not explained by a possible reaction to pan-allergens, such as profilin (Phl p 12) and polcalcin (Phl p 7), since only 27.5% of the patients contained serum sIgE against them. Group 1 is a major grass allergen ubiquitous in all grasses in contrast to group 5 which is absent in non-Pooideae grasses as is the case of CS. The data presented in this study strongly suggests that grass group 1 might be the culprit of the observed cross-sensitisation between autochthonous grasses (in this study, Phl) and CS.

Autochthonous grasses in Northern Spain flourish from April to July^1,16,17,26^, while CS flourishes from August to October^5,7^. From a clinical point of view, most patients (78.6%) referred a late allergic symptoms reactivation around September–October coincident with a second, August-to-October, peak of grass pollen counts in the air. At present, there are no commercial extracts of CS for immunotherapy. However, the overall improvement of symptoms usually reported by allergic patients that were treated with conventional grass immunotherapy, during both pollination peaks, suggests that they might have been protected also to CS pollen. This in addition holds clinical interest to those regions where CS is autochthonous and possibly allergenic.

The implications of the results into public health-related issues are many and diverse. First, the results encourage the international community to run allergenic tests to CS and to biochemically characterise the reaction to CS. Second, the results suggest that CS might lengthen the grass allergy season in territories where CS has expanded, by causing a second later peak, additional to the peak due to the autochthonous grasses. To note, commercially available grass immunotherapy might be beneficial to patients allergic to CS worldwide. Third, given that CS is banned in many countries and states, since it is considered an invasive species^3^, a demonstrated impact on human health would encourage policy makers to run programmes for eradicating this plant in non-autochthonous areas. The results provide an example of the global effects that alien invasive species can have on human health.

## Supplementary Information

Supplementary Information.
