# Author Correction: Allergenicity to worldwide invasive grass *Cortaderia selloana* as environmental risk to public health

**DOI:** 10.1038/s41598-022-06163-1

**Published:** 2022-01-31

**Authors:** Fernando Rodríguez, Manuel Lombardero-Vega, Lucía San Juan, Leticia de las Vecillas, Sofía Alonso, Eva Morchón, Diego Liendo, Marta Uranga, Alberto Gandarillas

**Affiliations:** 1 Allergy Service, Hospital Universitario Marqués de Valdecilla (HUM), Valdecilla 25, 39008 Santander, Spain; 2 CMC R&D Department, ALK-Abelló S.A., Miguel Fleta 19, 28037 Madrid, Spain; 3 Institute for Research Marqués de Valdecilla, Ave Herrera Oria SN, 39011 Santander, Spain; 4 Allergy Service, Sierrallana Hospital, Torrelavega, Spain; 5 Department of Plant Biology and Ecology, University of the Basque Country (UPV/EHU), Bilbao, Spain; 6 Asociación Cantabria Sin Plumeros, Liaño, Spain; 7 INSERM, Occitanie, Montpellier, France

Correction to: *Scientific Reports* 10.1038/s41598-021-03581-5, published online 24 December 2021

In the original version of this Article, Manuel Lombardero‑Vega was incorrectly affiliated with ‘INSERM, Occitanie, Montpellier, France’. The correct affiliation is listed below.

CMC R&D Department, ALK-Abelló S.A., Miguel Fleta 19, 28037 Madrid, Spain.

Furthermore, the original version of this Article omitted an affiliation for Alberto Gandarillas. The correct affiliations are listed below.

Institute for Research Marqués de Valdecilla, Ave Herrera Oria SN, 39011 Santander, Spain.

INSERM, Occitanie, Montpellier, France.

The original Article has been corrected.

