# Supplementary material for: Allergenicity to worldwide invasive grass Cortaderia selloana as environmental risk to public health
Source: Sci Rep. 2021 Dec 24;11:24426. doi: 10.1038/s41598-021-03581-5 (PMC8709847; doi:10.1038/s41598-021-03581-5)
Supplement: Supplementary file 1 — Supplementary Information. [file 41598_2021_3581_MOESM1_ESM.pdf]

SCIENTIFIC REPORTS

Allergenicity to worldwide invasive grass Cortaderia selloana as environmental risk to public health

Rodríguez et al

Supplementary Material

Suppl Table I
IgE results

Patient	Total IgE (IU/ml)	sIgE, ImmunoCAP, kUa/L					sIgE, RAST, kUa/L	
		Phleum	Phl p 1	Phl p 5	Phl p 7	Phl p 12	*C.selloana*	*P. pratense*
E0416001	97,70	16,10	2,87	12,00	0,00	0,18	0,53	6,81
E0416002	181,00	42,10	50,00	0,00	0,00	0,02	>27	>27
E0416003	262,00	97,10	82,80	34,70	0,01	0,02	>27	>27
E0416004	22,80	53,60	55,40	44,80	0,01	0,06	1,24	>27
E0416005	67,50	0,76	0,86	0,00	0,01	0,07	0,57	0,67
E0416006	453,00	13,40	20,70	6,61	0,01	0,02	14,76	13,56
E0416007	198,00	43,70	23,20	8,66	6,83	2,27	6,25	>27
E0416008	152,00	80,50	39,90	30,80	0,02	0,03	20,37	>27
E0416009	49,90	8,07	5,60	3,58	0,00	2,40	11,48	5,44
E0416010	88,00	15,60	12,90	4,52	0,00	0,01	1,44	8,68
E0416011	17,90	8,94	3,05	3,64	0,00	0,01	1,67	5,00
E0416012	178,00	5,61	7,03	0,01	0,01	0,01	4,71	3,82
E0416013	596,00	>100	>100	78,50	23,90	0,02	>27	>27
E0416014	207,00	9,46	10,60	0,01	0,01	0,02	0,42	10,99
E0416015	251,00	70,50	21,80	30,60	11,40	0,01	8,75	>27
E0416016		3,32	1,99	0,19	0,01	0,20	5,14	2,70
E0416017	45,60	4,22	3,32	0,00	0,00	0,00	1,07	4,83
E0416018	57,70	10,20	3,15	4,63	0,00	0,00	1,36	6,23
E0416019	272,00	88,80	47,90	28,80	0,01	0,01	>27	>27
E0416020	28,90	1,90	3,20	0,00	0,00	0,00	1,08	3,52
E0416021	280,00	35,00	24,50	8,30	0,01	0,02	>27	>27
E0416022	125,00	15,20	4,57	3,94	0,01	0,01	0,65	7,40
E0416023	533,00	25,80	36,10	0,01	0,02	0,03	4,57	>27
E0416024	28,10	1,75	1,03	0,00	0,00	0,00	0,17	1,28
E0416025	308,00	97,50	>100	36,50	0,46	0,35	>27	>27
E0416026	82,10	14,80	8,81	2,97	0,00	0,01	2,14	10,67
E0416027	75,30	17,10	9,20	8,22	0,00	0,22	9,02	11,88
E0416028	37,50	11,20	3,67	6,52	0,00	0,00	6,01	6,02
E0416029	57,70	2,12	1,23	0,41	0,00	0,01	0,40	1,22
E0416030	200,00	44,20	19,70	20,80	0,01	0,01	4,36	23,07
E0416031	13,20	1,58	2,18	0,00	0,00	0,00	0,89	1,13
E0416032	122,00	80,70	48,60	16,10	9,52	0,01	16,91	>27
E0416033	101,00	38,40	25,60	11,90	0,00	0,02	11,23	21,68
E0416034	16,10	3,17	1,02	1,48	0,00	0,00	0,17	2,81
E0416035	90,80	28,90	15,90	8,72	0,00	0,00	1,40	20,14
E0416036	165,00	15,20	23,60	0,02	0,00	0,00	6,76	17,95
E0416037	588,00	38,30	26,10	0,07	0,00	0,02	0,64	3,31
E0416038	37,70	3,70	2,34	1,06	0,00	0,06	3,88	>27
E0416039	30,30	--	3,37	2,28	0,00	0,10	1,22	5,44
E0416040	379,00	10,10	11,10	0,02	0,02	0,03	7,80	9,02
E0416041	89,50	3,56	2,34	0,00	0,00	0,00	0,17	2,92
E0416042	115,00	17,30	6,83	3,96	0,76	0,02	6,00	8,30
E0416043	282,00	16,70	22,30	0,42	0,00	0,00	6,29	16,82
E0416044	35,00	13,30	5,03	5,67	0,00	0,00	1,59	7,65
E0416045	13,30	0,41	0,23	0,00	0,00	0,00	0,17	0,26
E0416046	214,00	73,20	14,20	62,70	0,00	1,02	3,50	>27
E0416047	350,00	--	1,00	0,35	0,01	0,01	0,73	1,38
E0416048	69,80	14,20	4,72	4,99	0,00	0,00	1,32	8,96
E0416049	50,10	7,80	5,21	1,02	0,00	0,02	5,45	5,56
E0416050	145,00	44,40	33,60	17,90	0,01	0,01	7,72	22,96
E0416051	276,00	60,00	26,40	41,60	0,01	1,14	15,34	25,64
E0416052	934,00	38,30	31,10	12,00	0,04	0,06	16,76	14,85
E0416053	153,00	54,10	13,10	39,70	0,01	6,07	7,49	17,05
E0416054	306,00	11,70	14,00	0,01	0,01	0,02	4,12	8,57
E0416055	102,00	3,24	2,43	0,07	0,00	0,01	0,44	2,26
E0416056	457,00	3,12	2,60	0,00	0,00	0,00	0,75	2,29
E0416057	267,00	6,58	5,64	0,00	0,00	0,03	0,75	5,49
E0416058	611,00	>100	>100	>100	0,00	0,64	>27	>27
E0416059	246,00	23,60	13,80	10,90	0,00	0,00	3,26	12,57
E0416060	2950,00	>100	>100	>100	0,22	0,37	>27	>27
E0416061	181,00	59,10	17,70	43,70	0,00	0,00	2,18	26,25
E0416062	17,60	3,31	0,83	1,86	0,00	0,04	0,43	3,17
E0416063	212,00	0,50	0,42	0,00	0,00	0,00	0,53	0,38
E0416064	24,80	5,34	3,49	2,43	0,00	0,00	0,78	4,12
E0416065	23,10	3,35	1,22	0,63	0,00	0,02	0,35	2,37
E0416066	179,00	38,50	31,20	8,74	0,00	0,00	8,70	20,00
E0416067	179,00	38,50	31,20	8,74	0,00	0,00	0,17	0,75
E0416068	284,00	45,10	27,30	12,10	0,00	0,27	10,96	14,48
E0416069	24,00	3,63	1,26	1,69	0,00	0,03	0,57	2,63
E0416070	41,10	6,29	3,91	1,10	0,00	0,00	2,55	4,38
E0416071	165,00	6,02	3,15	1,54	0,00	0,00	2,68	2,41
E0416072	187,00	55,90	37,60	19,80	0,24	1,93	>27	>27
E0416073	135,00	1,72	2,71	0,00	0,00	0,01	0,17	1,01
E0416074	176,00	4,75	4,54	0,04	0,01	0,01	12,54	3,43
E0416075	116,00	44,50	22,80	19,20	3,97	1,58	15,34	19,1
E0416076	58,90	15,00	4,61	8,80	2,75	0,22	1,24	7,76
E0416077	1062,00	32,90	39,30	0,03	0,03	6,76	17,64	15,05
E0416078	168,00	33,30	16,30	15,90	2,81	0,22	5,93	12,75
E0416079	106,00	1,48	0,78	0,00	0,00	0,01	0,71	1,33
E0416080	528,00	72,70	57,40	11,60	0,01	0,69	>27	26,02
E0416081	1066,00	48,20	19,30	6,88	>100	2,58	>27	>27
E0416082	528,00	31,80	0,77	1,24	1,57	0,94	15,52	9,70
E0416083	432,00	1,13	0,04	0,00	0,01	0,85	0,86	0,80
E0416084	104,00	19,70	8,69	5,75	3,72	0,00	7,53	10,79
E0416085	1956,00	>100	94,50	>100	0,09	9,87	8,96	>27
E0416086	109,00	22,80	20,10	0,00	0,00	0,86	4,59	20,84
E0416087	370,00	>100	36,20	90,20	0,01	0,01	6,56	>27
E0416088	1622,00	>100	>100	>100	1,04	2,33	>27	>27
E0416089	55,80	11,20	3,47	2,36	0,00	2,70	4,09	15,67
E0416090	57,40	8,14	6,15	3,87	0,00	0,02	2,57	5,18
E0416091	124,00	15,40	8,93	6,49	0,00	0,00	2,77	10,41
E0416092	74,60	16,50	17,20	0,17	0,00	0,00	3,80	14,71
E0416093	10,60	3,70	1,22	2,09	0,00	0,03	0,84	2,86
E0416094	11,80	2,45	0,37	1,27	0,00	0,08,	0,44	1,42
E0416095	23,10	8,92	5,15	3,52	1,17	0,01	1,46	4,15
E0416096	8,93	1,40	0,38	1,24	0,00	0,00	0,17	0,81
E0416097	34,60	12,70	11,50	1,02	0,00	0,01	5,84	6,04
E0416098	105,00	19,20	5,74	11,30	2,61	0,86	2,49	11,93

Negative reaction is highlighted in pink.
Phl p: *Phleum* antigen group.

Supplementary Table II
Contingency table for sIgE to CS vs SPT to CS

		SPT CS		
		Pos	Neg	Total
sIgE CS	Pos	85	6	91
	Neg	2	5	7
	Total	87	11	98

P value and statistical significance	
Test	Fisher's exact test
P value	0,0001
P value summary	***
One- or two-sided	Two-sided
Statistically significant (P < 0.05)?	Yes

Rodríguez et al

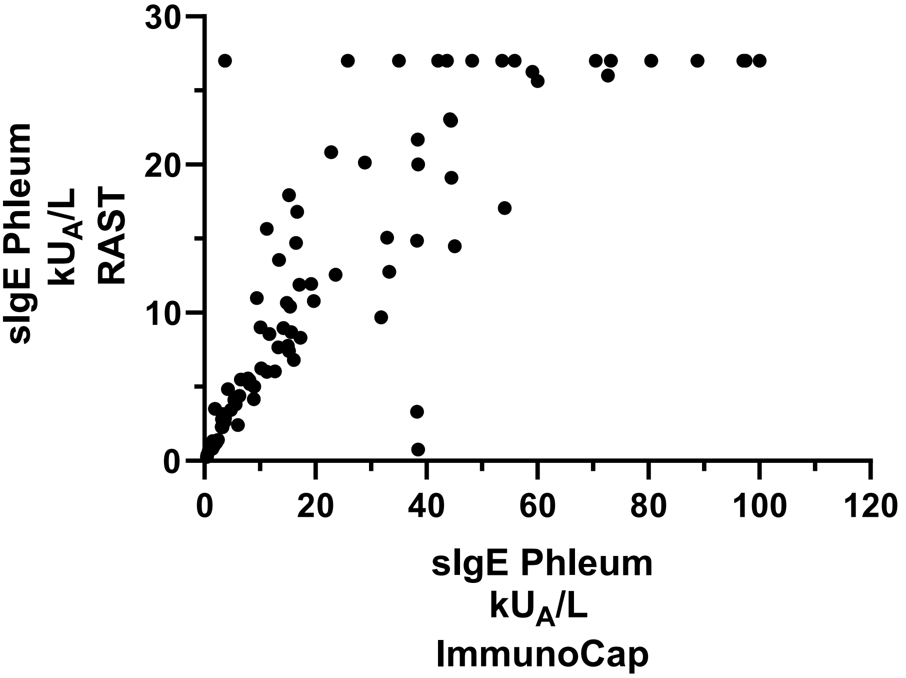

Spearman r	
r	0.8874
95% confidence interval	0.8337 to 0.9244
P value	
P (two-tailed)	<0.0001
P value summary	****
Exact or approximate P value?	Approximate
Significant? (alpha = 0.05)	Yes
Number of XY Pairs	96

Rodríguez et al

Supplementary Figure 1. Correlation between sIgE to *P. pratense* by RAST and by ImmunoCap

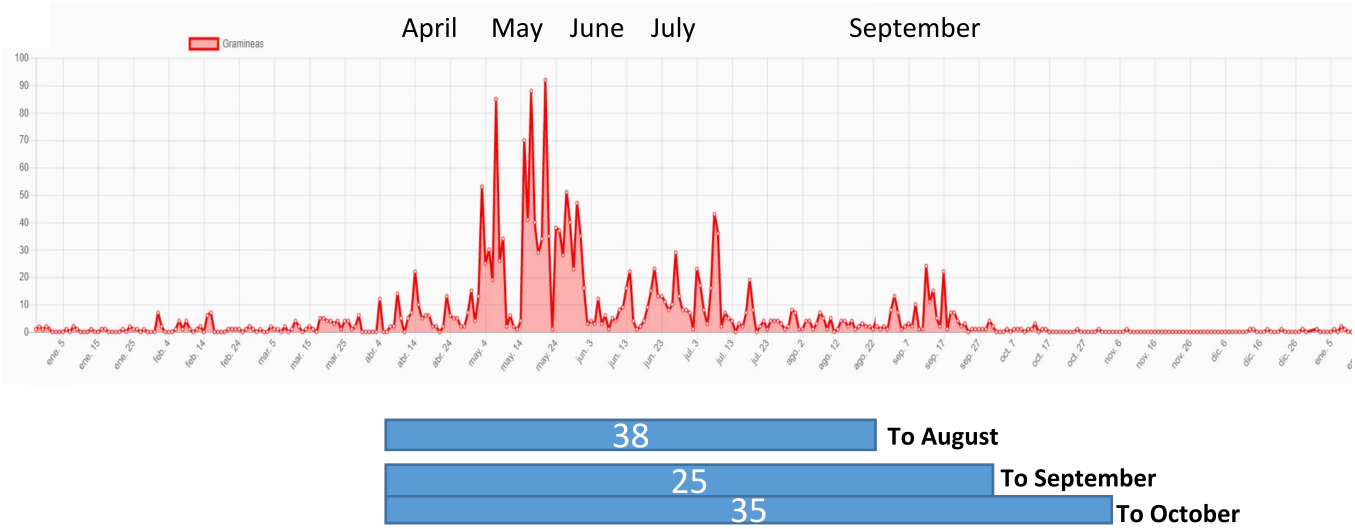

Rodríguez et al

Supplementary Figure 2. Concentration of grass pollen in the air along the year 2019. Data from the *Laboratorio de Salud Pública*, Gobierno Vasco, Bilbao. Concentration of grass pollen is compared to the periods when the patients reported allergic symptoms (blue bars). In white within the bars, the number of patients reporting symptoms up to August (when local grasses pollinise) to September or to October (when *Cortaderia Selloana* pollinises in the region).

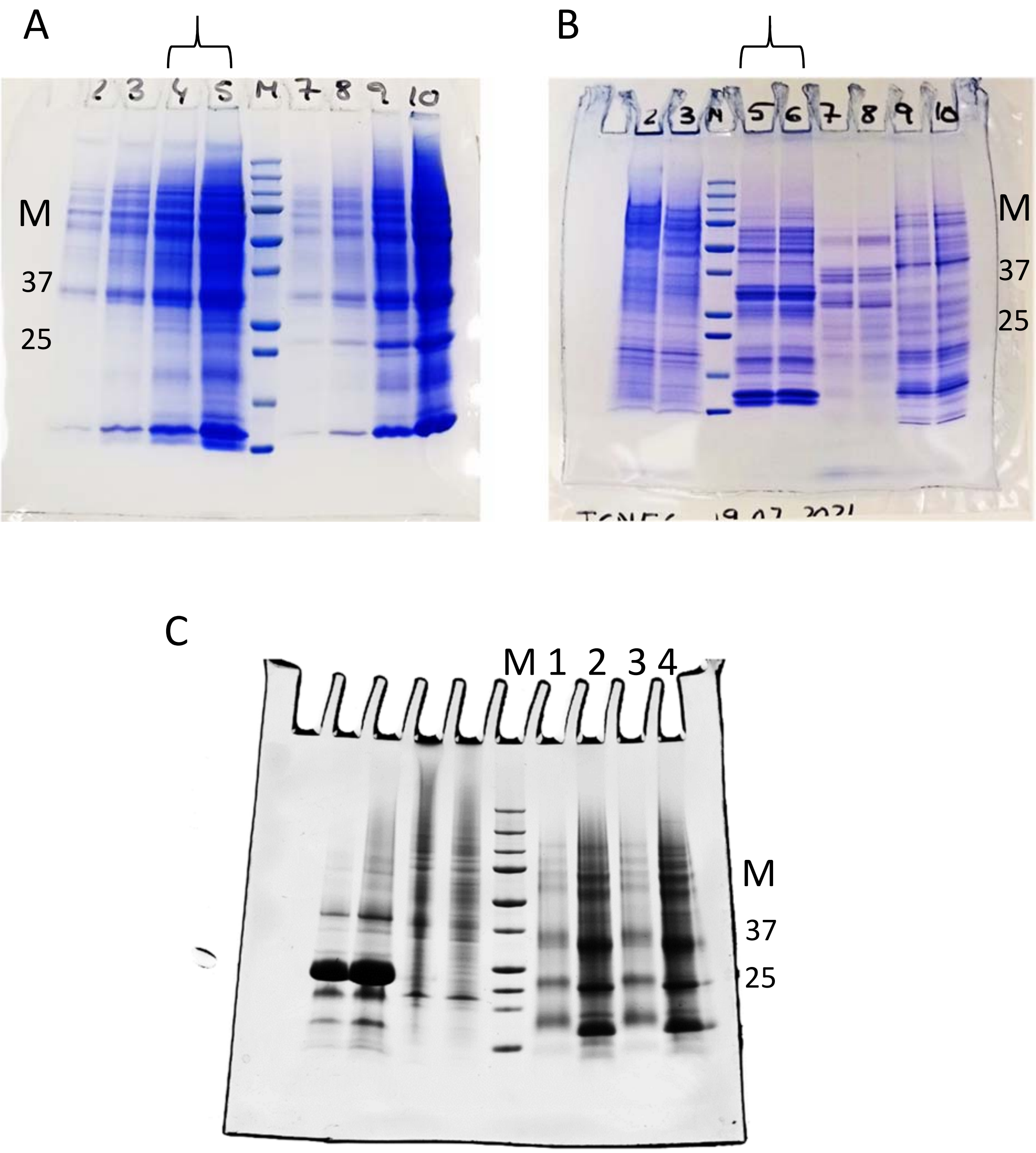

Rodríguez et al

Supplementary Figure 3. (A,B) Whole original gels to antigen 20/40 μl CS pollen extract (A; lanes 4,5) or 20 μl Plh pollen extract (B, lanes 5,6) shown in Figure 2A. (C) Similar antigen SDS-PAGE profiles for CS, 10/20 μl extract batches 07 (1,2) or 15 (3,4). M: Molecular weight markers (from bottom to top 10, 15, 20, 25, 37, 50, 75, 100, 150 and 250 kDa).
